# Spatial and temporal characteristics of gait as outcome measures in multiple sclerosis (EDSS 0 to 6.5)

**DOI:** 10.1186/s12984-015-0001-0

**Published:** 2015-02-10

**Authors:** Jana Lizrova Preiningerova, Klara Novotna, Jan Rusz, Lucie Sucha, Evzen Ruzicka, Eva Havrdova

**Affiliations:** Department of Neurology and Centre of Clinical Neuroscience, Charles University in Prague, First School of Medicine and General University Hospital, Katerinska 32, Prague 1, 128 00 Czech Republic; Department of Circuit Theory, Faculty of Electrical Engineering, Czech Technical University in Prague, Technicka 2, Prague 6, 166 27 Czech Republic

**Keywords:** Gait, Multiple sclerosis, EDSS, Velocity, Step length, GAITRite® instrument

## Abstract

**Background:**

Gait impairment represents one of the most common and disabling symptom of multiple sclerosis. Quantification of the gait is an important aspect of clinical trials. In order to identify which temporal or spatial parameters of gait could be used as outcome measures in interventional studies of patients with different levels of disability, we evaluated characteristics of these parameters in MS patients across the whole spectrum of mobility from EDSS 0 to 6.5.

**Methods:**

This is a cross-sectional study of spatial and temporal parameters of gait at self selected speed and at fast speed of walking in 284 patients with multiple sclerosis (108 men, mean age 38 years ± SD 10.8 years, range 18–64) divided into seven levels of disability (EDSS 0 to 1.5, EDSS 2.0 to 2.5, EDSS 3.0 to 3.5, EDSS 4.0 to 4.5, EDSS 5.0 to 5.5, EDSS 6.0, EDSS 6.5).

**Results:**

The velocity of gait decreases with increasing EDSS levels. Hovewer, the spatio-temporal parameters of gait that are involved in this process differ across the EDSS levels. The step length is decreased at higher EDSS levels up to the EDSS 6.0, but was not different between EDSS 6.0 and 6.5. The step time is significantly longer at EDSS 6.0 and 6.5, while the step length remains the same at those levels. The increase in percentage of double support time becomes statistically significant at EDSS 3.0-3.5 and continues to increase until EDSS 6.5. Variability of step time, step length or step width did not show significant difference between studied EDSS levels.

**Conclusions:**

There is no single spatio-temporal parameter of gait (other than velocity of gait) that would show significant differences among all levels of EDSS. The step length reflects shortening of steps at lower EDSS levels (2.0 to 6.0), and percentage of double support time better reflects changes at higher EDSS levels 3.0 – 6.5. Gait variability is not associated with disability in MS and therefore would not be a suitable outcome measure. These observations have to be considered when designing gait experiments with temporal and spatial parameters of gait as outcomes.

## Introduction

Over 75% of multiple sclerosis (MS) patients experience difficulty in walking [1]. Gait impairment can occur in all stages of MS and represents one of the most common and disabling symptom of this disease [2,3]. Quantifying impairment of walking is important when assessing new interventions to treat MS. The expanded disability scale score (EDSS) is the most common measure of disability used in multiple sclerosis studies, and although it is driven in part by ability to walk, it is limited in its sensitivity to measure gait impairment [4,5]. Other methods that have been validated to quantify ability to walk in MS patients, such as the 25 foot walk test (25FWT), two minute walk test, or patient reported Multiple Sclerosis Walking Scale (MSWS-12), do not allow assessment of the gait cycle [6,7]. It has been established that patients with MS have significantly different parameters of gait cycle when compared with healthy controls.

The most typical change in MS patients is a decrease in distance and speed of walking [8-11], but MS patients also have decreased stride length and limited range of ankle, knee and hip motion [10,11]. Prior studies investigating specifically spatial and temporal parameters of gait have reported decrease in velocity, cadence, step length, and increased step time in MS patients [2,8,12]. Martin et al. has shown also a trend towards increase in double support time in MS patients in comparison with normal controls [3]. Current studies provide an insight into gait changes in MS but do not provide enough of data to characterize gait cycle abnormalities at all levels of disability. In order to choose, which temporal or spatial parameters of gait are applicable as outcome measures at different levels of disability, we evaluated characteristics of these parameters in MS patients across the whole spectrum of mobility from EDSS 0 to 6.5.

## Methods

### Subjects

The study group in this observational cross-sectional study consists of 284 patients (108 men (38%)) with multiple sclerosis (mean age 38.9 years ± standard deviation (SD) 10.8 years, range 18–64) (Table 1). Consecutive patients whether treated or untreated were invited to participate in the study during the regular outpatient visits at the multiple sclerosis clinic affiliated with an academic institution. Patients with acute MS relapse, orthopaedic problems, or a vision problem severe enough to affect walking were excluded. EDSS scoring was performed by an MS specialist.

**Table 1:** **Patients characteristics across EDSS levels**

**EDSS**		**0-1.5**	**2.0-2.5**	**3.0-3.5**	**4.0-4.5**	**5.0-5.5**	**6**	**6.5**
Number of subjects	N	62	71	43	43	20	31	14
Female	%	64	55	76	67	75	45	43
Age	Mean ± SD	38.5 ± 9.3	34.1 ± 8.5	38.7 ± 9.1	40.2 ± 7.6	51.6 ± 9.7	46.9 ± 9.1	49.0 ± 12.9
	Range	18–57	20–58	24–61	24–60	34–64	23–64	30–64
Leg length (cm)	Mean ± SD	89.0 ± 5.3	90.0 ± 5.7	89.0 ± 6.0	90.0 ± 4.9	89.0 ± 4.1	90.0 ± 6.0	90.0 ± 5.2
	Range	79–102	78–105	78–102	79–102	82–98	78–102	83–98
Disease duration (years)	Mean ± SD	7.5 ± 4.9	8.1 ± 5.9	10.5 ± 6.8	14.3 ± 5.9	21.9 ± 10.9	21.7 ± 8.6	18.9 ± 10.5
	Range	1–20	1–27	2–31	2–33	7–45	3–37	2–39
Pyramidal subscore	Mean ± SD	0.77 ± 0.13	1.61 ± 0.52	2.30 ± 1.26	2.87 ± 0.35	3.15 ± 0.37	3.43 ± 0.57	3.40 ± 0.51
	Range	0–1	0–2	1–3	2–3	3–4	2–4	3–4
Cerebellar subscore	Mean ± SD	0.13 ± 0.34	0.40 ± 0.60	1.26 ± 0.88	1.86 ± 0.68	1.75 ± 0.55	1.93 ± 0.64	2.27 ± 0.96
	Range	0–1	0–2	0–3	0–3	1–3	0–3	0–4
Brainstem subscore	Mean ± SD	0.07 ± 0.25	0.28 ± 0.56	0.44 ± 0.77	1.00 ± 1.05	0.90 ± 0.91	0.90 ± 1.16	1.53 ± 1.25
	Range	0–1	0–2	0–3	0–3	0–3	0–3	0–3
Sensory subscore	Mean ± SD	0.56 ± 0.50	1.46 ± 0.63	1.91 ± 0.57	2.30 ± 0.74	2.35 ± 0.88	2.43 ± 0.86	2.47 ± 0.64
	Range	0–1	0–2	1–3	0–4	1–4	0–4	1–3

Ethic statement: The study procedures were approved by the institutional ethics committee of the General University Hospital and patients provided written informed consent.

### Gait analyses

The GAITRite**®** instrument (CIR systems Inc., Sparta, NJ) used to capture parameters of gait is a 16 feet (4.88 m) long carpet with pressure sensors that automates measurement of spatial (distance) and temporal (timing) characteristics of gait [13-15]. We tested gait at two different speeds. Patients were first instructed to walk two times the length of the carpet in their regular, self selected speed (SW), and then were asked to walk two times the length of the carpet as fast as they can (FW). Patients started from a standing position at the beginning of the carpet and walked without shoes (unless they used an ankle foot orthosis) without stopping past the end of the instrument.

Patients were divided into seven levels of disability based on the Kurtzke Expanded Disability Scale (EDSS) score [4]. The EDSS is not a continuous scale. The steps at the lower end of the scale, such as EDSS 1.0 to 3.5 are calculated as a composite score of seven functional systems (pyramidal, cerebellar, brainstem, sensory, bowel and bladder, visual and cerebral score). The scores at and above 4.0 are determined mostly by a maximal distance reached without support (4.0 = 500 m, 4.5 = 300 m, 5.0 = 200 m and 5.5 = 100 m). The EDSS 6.0 is primarily defined by a need of unilateral aid for walking of at least 100 m and the EDSS level 6.5 is defined by a need of bilateral walking aid. As the half step on EDSS scale at EDSS levels 1.0 to 5.5 does not represent significant difference in disability, for the purpose of our analysis, we defined the following groups: EDSS score 1.0 and 1.5 in one group, and similarly EDSS 2.0-2.5, EDSS 3.0-3.5 as well as EDSS 4.0-4.5 and EDSS 5.0-5.5 into separate groups. Since there is a major difference between EDSS 6.0 and 6.5 (unilateral versus bilateral walk aid), these groups are analysed as two separate levels of disability.

### Statistics

Descriptive statistics were used to characterize the population. The Kolmogorov-Smirnov test for independent samples was used to test for normality of the distribution in all parameters. As a result, GaitRite provides more than 40 variables for each leg and a number of these variables are related one to another. Therefore, to find relevant measurements in order to assess the extent of gait impairment and to avoid multiple comparisons, we have first applied simple pre-selection stage. The Pearson correlation coefficient was used to identify highly correlated measurements (*r* > 0.9) and to select representative variables for further analyses.

Subsequently, a two-way repeated measures analysis of covariance (ANCOVA) was used to assess the effect of factors involving Group (EDSS 0–1.5, 2.0-2.5, 3.0-3.5, 4.0-4.5, 5.0-5.5, 6, 6.5) and Walking (SW, FW), and interactions between these two factors, with age and leg length set as covariates. Post-hoc Bonferroni adjustment was then applied to determine differences between individual EDSS groups and walking conditions. A nominal alpha level of 0.05 was used for all comparisons. The main effects and interactions resulting from ANCOVA were interpreted as follows: (a) a main effect of Group (meaning EDSS group) is statistically significant if there is difference in patients' performance across individual EDSS groups, i.e. patients with higher EDSS scores generally reached worse performance than those with lower EDSS scores; (b) a main effect of Walking (SW versus FW) is statistically significant if the measured parameter reflects speed of walking; (c) an interaction involving Group x Walking is statistically significant if the measured parameter is dependent on the speed of walking, i.e. difference between fast and normal speed of walking. Post-hoc comparisons were used to evaluate whether the individual EDSS groups can be separated across tested parameters.

## Results

**Figure 1:**
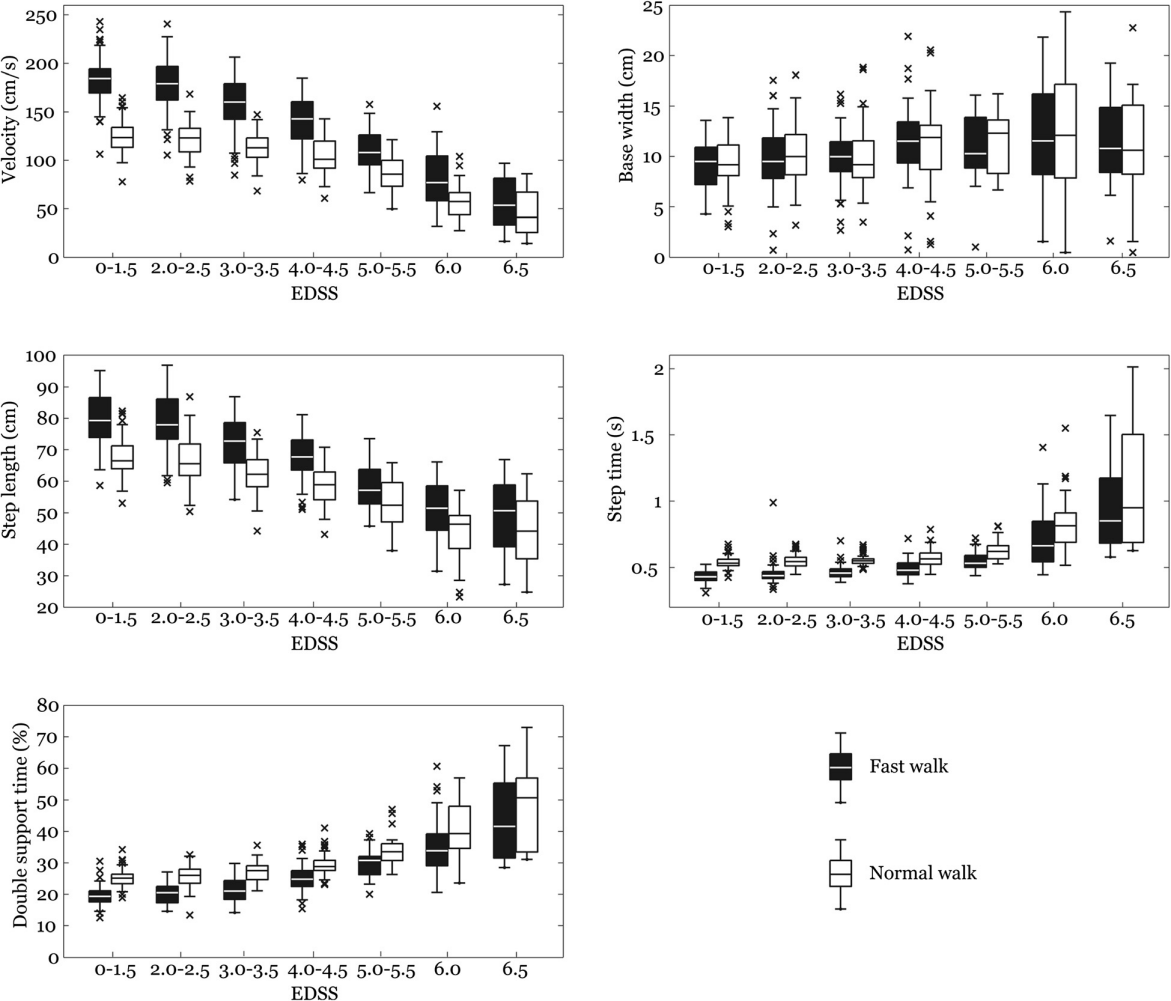
**Comparison of gait measures for self-selected and fast walk speed across different EDSS levels.**

The Kolmogorov-Smirnov test indicated that gait variables were normally distributed across EDSS groups. Using Pearson correlation, we have identified five representative variables including velocity, step length, step time, double support time, and base width; the correlation coefficient between these variables was not greater than 0.9 (r = 0.12–0.88, *p* < 0.01). Since the results of the left and right leg for investigated variables were highly correlated (r = 0.91–0.96, *p* < 0.001), we further analysed data based only on the left leg. In addition, when applicable, the variability of investigated parameters were also included in the analyses (i.e., step length SD, step time SD, and support base SD). The description of variables used in the current study can be found in Table 2. The results for the seven EDSS groups in both walking conditions (SW, FW) across each investigated parameter are interpreted using mean ± SD (Table 3) as well as boxplots (Figure 1). In addition, Table 4 lists mean ± SD of the variability of gait parameters.

**Table 2:** **Definition of outcome measures**

**Outcome**	**Description**
Step length	Distance from a point of the ground contact of one foot to the point of the ground contact of the other foot, i.e. left to right foot and right to left foot.
Step time	Period of time taken for one step.
Percentage of double support time	A proportion of double support time from the gait cycle. Double support time is a period of time when both feet are in contact with the ground. It occurs twice in the gait cycle, at the beginning and at the end of the stance phase.
Base width	Lateral distance between the heel centre of one foot to the line of progression of the other side.

**Table 3:** **Gait parameters across EDSS levels**

**EDSS levels**	**Walking conditions**	**Velocity (cm/s)**	**Step length (cm)**	**Step time (s)**	**Double support time (%)**	**Base width (cm)**
0-1.5	Normal	124.9 ± 17.3	67.7 ± 6.4	0.54 ± 0.05	25.2 ± 2.8	9.3 ± 2.6
	Fast	182.6 ± 24.6	79.6 ± 8.2	0.43 ± 0.04	19.5 ± 3.1	9.2 ± 2.5
2.0-2.5	Normal	120.8 ± 17.6	67.4 ± 7.1	0.55 ± 0.05	25.6 ± 3.3	10.2 ± 2.9
	Fast	178.4 ± 25.5	79.0 ± 9.3	0.45 ± 0.08	20.2 ± 3.3	9.8 ± 3.1
3.0-3.5	Normal	112.7 ± 16.0	62.2 ± 6.8	0.55 ± 0.04	27.1 ± 3.1	9.8 ± 3.2
	Fast	155.3 ± 29.9	72.4 ± 8.8	0.47 ± 0.06	21.6 ± 3.7	9.8 ± 3.0
4.0-4.5	Normal	103.4 ± 19.0	58.5 ± 6.4	0.57 ± 0.07	29.4 ± 3.9	11.1 ± 4.1
	Fast	139.6 ± 25.4	67.3 ± 7.1	0.49 ± 0.07	25.1 ± 4.5	11.3 ± 3.9
5.0-5.5	Normal	85.5 ± 18.5	52.9 ± 7.8	0.63 ± 0.09	34.2 ± 5.5	12.1 ± 4.3
	Fast	109.9 ± 24.5	58.5 ± 7.3	0.55 ± 0.08	29.8 ± 5.0	11.6 ± 5.2
6.0	Normal	57.3 ± 19.1	43.5 ± 9.0	0.82 ± 0.23	41.2 ± 8.0	12.2 ± 6.0
	Fast	79.5 ±	50.8 ± 10.4	0.73 ± 0.24	35.6 ± 9.5	12.2 ± 5.5
6.5	Normal	47.5 ± 24.4	45.0 ± 11.3	1.09 ± 0.46	48.4 ± 14.9	11.0 ± 5.9
	Fast	58.2 ± 26.7	49.4 ± 11.9	0.95 ± 0.36	44.6 ± 13.5	11.4 ± 4.7
Overall	Normal	104.8 ± 30.4	60.4 ± 11.0	0.62 ± 0.19	29.8 ± 8.4	10.5 ± 3.9
	Fast	148.4 ± 47.2	70.4 ± 13.8	0.52 ± 0.18	24.6 ± 8.8	10.4 ± 3.8

The results are expressed as mean ± SD.

**Table 4:** **Variability of gait measures across EDSS levels**

**EDSS levels**	**Walking conditions**	**Step length variability (cm)**	**Step time variability (ms)**	**Base width variability (cm)**
0-1.5	Normal	2.5 ± 1.4	20.4 ± 12.2	2.06 ± 0.94
	Fast	2.8 ± 1.5	20.3 ± 16.5	2.12 ± 1.05
2.0-2.5	Normal	2.7 ± 1.5	20.4 ± 14.5	2.13 ± 1.03
	Fast	3.2 ± 1.8	38.2 ± 13.2	2.13 ± 1.15
3.0-3.5	Normal	2.7 ± 1.4	23.5 ± 18.7	2.13 ± 1.03
	Fast	3.0 ± 1.3	28.9 ± 82.9	2.83 ± 1.53
4.0-4.5	Normal	2.9 ± 1.2	30.5 ± 30.6	2.98 ± 1.75
	Fast	3.0 ± 1.4	37.7 ± 116.9	2.83 ± 1.31
5.0-5.5	Normal	4.1 ± 2.5	43.3 ± 31.6	2.98 ± 1.52
	Fast	4.4 ± 2.5	33.0 ± 25.3	3.38 ± 2.36
6.0	Normal	4.8 ± 3.5	93.8 ± 106.1	3.51 ± 3.26
	Fast	3.9 ± 2.0	58.0 ± 47.9	3.15 ± 2.72
6.5	Normal	3.0 ± 0.8	86.4 ± 72.1	2.81 ± 1.28
	Fast	5.4 ± 5.2	74.5 ± 58.9	2.50 ± 1.19
Overall	Normal	3.0 ± 1.9	35.2 ± 48.8	2.48 ± 1.62
	Fast	3.3 ± 2.1	36.5 ± 90.1	2.55 ± 1.60

The results are expressed as mean ± SD.

### Velocity

Velocity of gait significantly decreased with increasing disability level during both SW and FW tests. Results of the ANCOVA for velocity showed significant main effects for Group [F(6, 569) = 126.1, *p* < 0.001, *η*^2^ = 0.58] and Walking [F(1, 569) = 280.5, *p* < 0.001, *η*^2^ = 0.34], as well as a significant interaction effect for Group × Walking [F(6, 569) = 11.1, *p* < 0.001, *η*^2^ = 0.11]. Post-hoc comparisons revealed significant differences between any two consecutive disability groups through EDSS 2.0 to 6.5 (2.0-2.5 *vs*. 3.0-3.5: *p* = 0.001; 3.0-3.5 *vs*. 4.0-4.5: *p* = 0.005; 4.0-4.5 *vs*. 5.0-5.5: *p* = 0.001; 5.0-5.5 *vs*. 6.0: *p* < 0.001; 6.0 *vs*. 6.5: *p* = 0.046). The differences in velocity between the two lowest Groups (EDSS 0–1.5 vs. 2.0-2.5) did not reach statistical significance. The difference in velocity between SW and FW continuously diminished with increasing disability. There was no difference in velocity between SW and FW at EDSS 6.5.

### Step length

Step length significantly decreased with increasing disability when comparing between any two consecutive Groups from EDSS 0 to 6.0 in both SW and FW. There was no significant difference in step length between EDSS 6.0 and 6.5. Analysis of step length revealed a significant main effects for Group [F(6, 569) = 91.0, *p* < 0.001, *η*^2^ = 0.50] and Walking [F(1, 569) = 138.1, *p* < 0.001, *η*^2^ = 0.20], as well as a significant interaction effect for Group × Walking [F(6, 569) = 2.6, p = 0.02, *η*^2^ = 0.03]. Post-hoc comparisons revealed significant differences between EDSS groups 2.0-6.0 (2.0-2.5 *vs*. 3.0-3.5: *p =* 0.001; 3.0-3.5 *vs*. 4.0-4.5: *p =* 0.002; 4.0-4.5 *vs*. 5.0-5.5: *p =* 0.01; 5.0-5.5 *vs*. 6.0: *p* < 0.001). There were no significant differences between the two lowest and between the two highest Groups (EDSS 0–1.5 *vs*. 2.0-2.5 and 6.0 *vs*. 6.5). Only at EDSS 6.5 there was no significant difference in step length between FW and SW.

Statistical analysis of step length SD (indicating variability of step length) revealed a significant main effects for Group [F(6, 569) = 32.7, *p* < 0.001, *η*^2^ = 0.09] and Walking [F(1, 569) = 19.1 *p* = 0.02, *η*^2^ = 0.01], as well as a significant interaction for Group × Walking [F(6, 569) = 5.3, p = 0.02, *η*^2^ = 0.03]. Post-hoc comparisons revealed significant differences only between two consecutive EDSS groups 4.0-4.5 *vs*. 5.0-5.5 (*p =* 0.003).

### Step time

Step time was significantly shorter when comparing any two consecutive Groups between EDSS 5.0 to 6.5 in both SW and FW, but not for patients with EDSS below 5.0. There was no significant difference in step time between FW versus SW in patients who reached EDSS 6.0 and 6.5. Step time showed significant main effects for Group [F(6, 569) = 79.8, *p* < 0.001, *η*^2^ = 0.46] and Walking [F(1, 569) = 61.9, *p* < 0.001, *η*^2^ = 0.10], but no significant interaction for Group × Walking [F(6, 569) = 0.3, *p* = 0.96, *η*^2^ = 0]. Post-hoc analysis showed no significant differences in step time within EDSS 0–5.5, while there is high statistically significant difference between groups with EDSS 5.0-6.5 (5.0-5.5 *vs*. 6.0: *p* < 0.001; 6.0 *vs*. 6.5: *p* < 0.001).

Statistical analysis of step time SD (indicating variability of step time) showed significant main effects for Group [F(6, 569) = 6.0, *p* < 0.001, *η*^2^ = 0.06] but no significant effect for Walking [F(1, 569) = 0.3, *p* = 0.56, *η*^2^ = 0] or interaction Group × Walking [F(6, 569) = 1.2, *p* = 0.37, *η*^2^ = 0.01]. Post-hoc analysis did not revealed any significant differences between two consecutive groups.

### Percentage of double support time

The percentage of double support time is increased in patients with higher disability score, when comparing any two consecutive Groups from EDSS 4.0 to 6.5 in both SW and FW, but not for patients with EDSS below 4.0. Analysis of the double support time showed significant main effects for Group [F(6, 569) = 102.6, *p* < 0.001, *η*^2^ = 0.53] and Walking [F(1, 569) = 93.7, *p* < 0.001, *η*^2^ = 0.15], but no significant interaction for Group × Walking [F(6, 569) = 0.3, *p* = 0.94, *η*^2^ = 0]. Post-hoc analysis revealed significant differences in moderate-to-higher EDDS groups (3.0-3.5 *vs*. 4.0-4.5: *p* = 0.02; 4.0-4.5 *vs*. 5.0-5.5: *p* = 0.02; 5.0-5.5 *vs*. 6.0: *p* < 0.001; 6.0 *vs*. 6.5: *p* < 0.001), whereas no significant differences were found between low-to-moderate EDSS levels (0–3.5).

### Base width

Patients with higher EDSS scores generally did not have significantly increased base width. Statistical analysis showed significant effect for Group [F(6, 569) = 4.8, *p* < 0.001, *η*^2^ = 0.05], but not for Walking [F(1, 569) = 0.01, *p* = 0.91, *η*^2^ = 0] or the interaction Group × Walking [F(6, 569) = 0.12, *p* = 0.99, *η*^2^ = 0]. However, post-hoc analysis did not revealed any significant differences between two consecutive groups.

Statistical analysis of base width SD (for variability of base width) showed significant effect for Group [F(6, 569) = 13.3, *p* < 0.001, *η*^2^ = 0.06], but not for Walking [F(1, 569) = 0.11, *p* = 0.74, *η*^2^ = 0] or the interactions Group × Walking [F(6, 569) = 1.0, *p* = 0.41, *η*^2^ = 0.1]. Post-hoc analysis did not revealed any significant differences between two consecutive groups.

## Discussion

In this study of 284 MS subjects with EDSS 0 – 6.5, we focused on analysis of the gait cycle to understand changes that occur at different levels of disability and how these parameters could be used as outcome measures. The decrease in step length is a phenomenon observed at EDSS levels 0–6.0 that reaches a plateau between EDSS 6.0 and 6.5.

The other phenomenon we observed is the prolongation of the step time, which reached significance at EDSS 5.0 and higher. The velocity at EDSS 6.0 and 6.5 is significantly lower, however the step length remained constant. The extra portion of the step time is spent in the double support phase of the gait cycle. The increase in percentage of double support time becomes statistically significant at EDSS 3.0-3.5 and continues to increase in all groups until EDSS 6.5.

Although the velocity was not the prime focus of our study, assessing it at individual EDSS levels had shown interesting observations. Patients with EDSS 0–3.5 are all defined as fully ambulatory by EDSS definition, however patients at EDSS3.0-3.5 walk significantly slower than patients with EDSS 0–1.5 and 2.0-2.5. When comparing velocity at SW versus FW, we observed that patients with EDSS 3–3.5 had a significantly lower capacity to increase velocity between SW and FW than patients with EDSS 0–1.5 and 2.0- 2.5 (Figure 1). This phenomenon is even more revealing in patients with higher EDSS levels. Velocity of patients with EDSS 6.0 at SW was about half of the velocity of patients with EDSS 3.0 -3.5 (57 cm/s vs. 112 cm/s). The difference in velocity between FW and SW at EDSS 3.0-3.5 was 43 cm/s, while patients with EDSS 6.0 were able to increase their speed only by 22 cm/s. Thus, patients with higher EDSS levels were not only walking slower, but their velocity at SW was much closer to their maximal speed of walking than in patients with lower EDSS. Patients at EDSS 6.5 seem to be unable to increase the speed between SW and FW tests, so they walk at their maximal speed of walking at all times (Figure 1). The relationship between SW and FW in MS has not been widely studied, as MS studies use mostly fast walk tests. However, Van der Linden et al. had shown that MS patients walking at self selected speed had reduced dorsiflexion at initial contact with the floor, reduced plantar flexion and reduced knee flexion in swing phase as the possible mechanisms of speed decline in MS [16]. When we compare the velocity of patients in our study to the data from meta analysis of normal speed of gait by Bohannon, the velocity of 112.67 ± 15.95 cm/s reached at SW by patients with EDSS 3.0-3.5 (mean age 38.7, SD 9.1) corresponds to a self selected speed of gait typical in the normal population for women in the seventh decade (113.2 cm/s (range 107.2 to 119.2) [17].

Whereas our dataset is focused on assessment of gait characteristics at individual EDSS levels, the results are in general agreement with prior studies of gait abnormalities in MS, such as of Givon et al., who studied group of 81 independently walking MS patients (mean EDSS 2.8, range 0–5.5) and documented that these patients walked slower, used shorter steps, had longer step time than normal controls and the gait parameters correlated strongly with EDSS scores [18]. Sosnoff et al., had also shown the prolongation of double support time in 13 MS patients with EDSS 4.0-6.0 compared with normal controls [19]. Soccie et al. in 2012 had shown in a study that focused on gait variability in MS (N = 88) that individuals who used assistive devices for ambulation (EDSS 6.0-6.5) had significantly lower velocity of gait and shorter step length than independent walkers (EDSS 2.0-5.5) [20].

We did not find statistical difference in the base width across the EDSS levels. Prior studies have demonstrated that MS patients in general have wider base of gait than normal controls, however they did not show significant correlation between EDSS and base width [20]. Contrary to that, Balantrapu et al. have observed that patients with higher EDSS have more spasticity, which correlated with a decrease in base width [21]. We did not evaluate spasticity in lower extremities, which limits us in assessing the role of spasticity in base width.

Investigation of variability of gait, measured as step to step variation, did not reveal any significant difference between the EDSS levels. Stride to stride variation was shown to correlate with disease severity and response to treatment in diseases that involve basal ganglia (such as Parkinson disease and Morbus Huntington) [22]. Variability of gait in multiple sclerosis measured using GAITRite walkway over a short distance is not significant.

## Conclusions

This study elucidated how the temporal and spatial parameters of gait change across the levels of disability from EDSS 0 to 6.5. It is obvious that there is no single parameter (other than velocity of gait) that could be used to measure changes at all levels of disability. However, there are different parameters involved in velocity loss at different EDSS levels. The step length shortens significantly at EDSS 2.0 to 6.0, and percentage of double support time is significantly prolonged at EDSS 3.0 – 6.5. The shortening of step length plateaus at EDSS 6.0 – 6.5. Parameter that does not differ between EDSS 6.0 and 6.5 will not be useful outcome to measure gait changes at those levels. The cross-sectional nature of the study limits us in understanding how these variables would change in individual patients with progression of EDSS over time. Evaluation of walking at SW and FW in the same setting had shown us that the fast speed of walking is more sensitive to detecting patient’s deficits. These facts have to be considered when designing gait experiments with temporal and spatial parameters of gait as outcome measures.
